# Surgical hyoid bone repositioning effects on mandibular advancement and upper airway collapsibility: an anesthetized rabbit model study

**DOI:** 10.3389/fphys.2025.1618037

**Published:** 2025-11-17

**Authors:** Hiba J. Tannous, Corine J. Samaha, Hassan A. Chami, Joseph G. Ghafari, Jason Amatoury

**Affiliations:** 1 Sleep and Upper Airway Research Group (SUARG), American University of Beirut, Beirut, Lebanon; 2 Department of Dentofacial Medicine, American University of Beirut Medical Centre, Beirut, Lebanon; 3 School of Medicine, Johns Hopkins University, Baltimore, MD, United States; 4 Department of Orthodontics, University of Pennsylvania, Philadelphia, PA, United States; 5 Biomedical Engineering Program, Maroun Semaan Faculty of Engineering and Architecture (MSFEA), American University of Beirut, Beirut, Lebanon

**Keywords:** obstructive sleep apnea, OSA, upper airway surgery, oral appliance, mandibular advancement splint, closing pressure, pharynx, hyomandibular suspension

## Abstract

**Background:**

Mandibular advancement serves as a treatment option for obstructive sleep apnea (OSA), but its effectiveness differs among patients. The position of the hyoid bone is crucial for maintaining upper airway patency and may influence mandibular advancement outcomes. This study aimed to assess the impact of surgical hyoid re-positioning on mandibular advancement-induced changes in upper airway collapsibility in an animal model.

**Methods:**

Twelve anesthetized male New Zealand White rabbits underwent mandibular advancement (0–4 mm), combined with hyoid repositioning in various directions (anterior, cranial, caudal, anterior-cranial, anterior-caudal) and increments (0–4 mm). Upper airway collapsibility was quantified as the negative pressure required to close the airway (Pclose) at various mandibular and hyoid positions.

**Results:**

Increasing mandibular advancement alone led to a progressive reduction in Pclose, indicating a decrease in upper airway collapsibility. Similarly, anterior hyoid repositioning alone resulted in incremental reductions in Pclose, with similar outcomes observed for anterior-cranial and anterior-caudal directions. When mandibular advancement was combined with anterior-based hyoid repositioning directions, a further decrease in Pclose was observed compared to when either intervention was applied alone. Cranial and caudal hyoid repositioning had no direct effect on Pclose or on mandibular advancement outcomes.

**Conclusion:**

In summary, decreases in upper airway collapsibility induced by mandibular advancement are dependent on both hyoid repositioning direction and increment. The findings suggest that combining mandibular advancement with anterior-based hyoid repositioning may enhance the effectiveness of mandibular advancement in treating OSA.

## 1 Introduction

The hyoid bone and mandible are crucial in maintaining the patency of the upper airway. Abnormal positioning of these bones can affect the mechanical behavior of upper airway tissues (including how they deform) and the effectiveness of pharyngeal muscles in responding to both static and dynamic upper airway loads (e.g., change in mandible position, intraluminal pressure variations, muscle activity) (Bilston and Gandevia, 2014). Individuals with obstructive sleep (OSA) often exhibit an inferiorly positioned hyoid bone and a retruded mandible compared to healthy individuals (Ahmadi et al., 2022; Chi et al., 2011; Lee et al., 2009). These anatomical deviations are associated with a more collapsible upper airway, a characteristic feature of OSA (Chi et al., 2011; Neelapu et al., 2017; Bilici et al., 2018).

OSA is a highly prevalent disorder associated with serious health consequences, such as cardiovascular diseases and cognitive impairments (McEvoy et al., 2016; Young et al., 1993; Barletta et al., 2019). Accordingly, the treatment of OSA is a major health priority. Mandibular advancement, a treatment option for OSA that involves protruding the mandible using a dental appliance to keep the upper airway open during sleep, has been shown to reduce airway collapsibility (Bamagoos et al., 2019). The success of mandibular advancement is reported in approximately 50% of patients, but the reasons for this variability are not well understood (Sutherland et al., 2014).

The mandible is connected to the hyoid bone via several pharyngeal muscles, including the genioglossus, geniohyoid, mylohyoid and digastric muscles (Mu and Sanders, 2010; Edwards and White, 2011). As a result of the hyoid-mandible connections, a lower hyoid bone may decrease the effectiveness of mandibular advancement therapy due to alteration of muscle angles and/or altered tissue mechanical (stiffness) properties (Bilston and Gandevia, 2014; Salman and Amatoury, 2024). Hyoid repositioning surgeries, such as those involving anterior-cranial elevation of the hyoid to the mandible (hyomandibular suspension) or anterior-caudal hyoid attachment to the thyroid cartilage (hyothyroidopexy), are conducted to help compensate for the lower hyoid position in OSA and/or stabilize/enlarge the upper airway and improve clinical outcomes (Song et al., 2016; Baker et al., 2025). However, the combined effects of surgical hyoid repositioning and mandibular advancement on OSA treatment outcomes remain unclear, warranting further research to better understand this interaction and potentially improve therapeutic strategies.

The aims of this study were to investigate the impact of hyoid bone surgical repositioning and mandibular advancement, alone and in combination, on upper airway collapsibility, using an anaesthetized rabbit model. Rabbits were selected as an ideal model due to their fundamentally comparable upper airway anatomy and physiology to humans, including having a freely suspended hyoid bone (Amatoury et al., 2014; Amatoury et al., 2015), which differs from most non-primates in which the hyoid bone is relatively fixed (Van de Graaff et al., 1984).

## 2 Methods

Studies were performed on 12 adult, male, New Zealand White rabbits weighing 2.9 ± 0.9 kg and approximately 6 months of age. The rabbits were bred and housed in the animal care facility at the American University of Beirut. All rabbits included in the study were healthy adult animals of the same species, gender, and similar age, with no prior experimental treatments. Each rabbit was isolated and fasted for approximately 12 h the night before experimentation, The protocol was approved by the Institutional Animal Care and Use Committee at the American University of Beirut (#19-08-544).

### 2.1 Experimental setup

**FIGURE 1:**
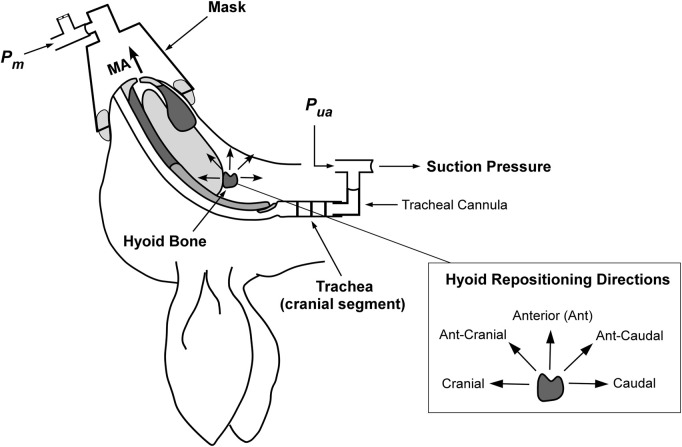
Schematic of the anaesthetized supine rabbit model. The upper airway is isolated at the level of the trachea. Mandibular advancement (MA) is applied in set increments. Hyoid displacement is applied to reposition the hyoid in the indicated directions (shown inset on right). Suction pressure (applied with a syringe) at the caudal tracheal end for closing pressure determination using upper airway pressure (Pua) and mask pressure (Pm). Figure adapted and modified from Samaha et al. under the CC-BY license (Samaha et al., 2022).

Most of the experimental methodology, except for that associated with mandibular advancement, has been previously reported (Samaha et al., 2022). The experimental setup is illustrated in Figure 1.

The rabbits were anesthetized with an intramuscular injection of ketamine (35 mg/kg) and xylazine (5 mg/kg) followed by a continuous intravenous infusion of ketamine (15 mg/kg/hr) and xylazine (4.5 mg/kg/hr) to maintain anesthesia throughout the experiment. Heart and respiratory rates were monitored to ensure the rabbits’ physiological stability. At the conclusion of the experiment, the rabbits were euthanized using an anesthetic overdose.

The rabbits were positioned supine on a surgical platform. The head/neck position was controlled, such that a line drawn from the tragus to the external nares was at 50° to the horizontal. A ventral skin incision was made on the neck and blunt dissection was performed to expose the trachea. The baseline position of the trachea, taken between the third and fourth tracheal cartilage rings, was marked on the fixed experimental platform at the end of expiration.

The trachea was completely transected between the third and fourth tracheal rings to isolate the upper airway. This procedure resulted in the absence of airflow though the upper airway and the rabbits breathed spontaneously via the caudal trachea. An L-shaped tube was inserted into the caudal trachea and the pressure was monitored via this connection using a pressure transducer (Validyne DP45–32; Validyne Engineering, Northridge, CA). Another L-shaped tube was secured into the cranial trachea to reposition the tracheal segment to its pre-transection baseline position. A calibrated syringe and 100 cm volume extension for upper airway pressure application, and a pressure transducer (Validyne DP45–32) for measuring upper airway pressure (Pua), were also connected to the cranial L-shaped tube.

A small modified conical animal anesthetic mask (GaleMed VM-2, GaleMed, Taiwan) with inflatable sleeve was fitted to the rabbit’s snout to achieve a closed upper airway system. The mask allowed for a sealed system and the application of upper airway intra-luminal pressure and measurement of mask pressure (Pm) via a pressure transducer (Validyne DP45–32). To ensure a complete mask seal, the system was pressurized using a syringe. Pressure leaks were eliminated using petroleum jelly around the mask and inflation of the mask sleeve.

All surgical procedures, interventions, and measurements (see below) were performed by the same trained researchers using standardized protocols and instrumentation. Although intra-rater variability was not formally assessed, consistency across animals was ensured through the use of uniform techniques and close coordination among the operators.

### 2.2 Hyoid repositioning

A hyoid bone repositioning device was developed in-house to displace the hyoid bone in various increments and directions, as previously described (Samaha et al., 2022). Briefly, to attach the hyoid bone to the repositioning device, a miniscrew (RMO® Dual-Top, 2 mm × 8 mm) was inserted into the central part of the body of the hyoid. The device consisted of a horizontal sliding platform positioned above the rabbit. A modified digital caliper with a rigid extension and alligator clamp was mounted perpendicular to this platform and connected to the hyoid miniscrew. This caliper was used to advance the hyoid anteriorly by set increments and then return it to baseline. The sliding platform itself could move in the cranial–caudal direction, with these displacements measured using a second digital caliper. By adjusting both calipers, combined anterior–cranial or anterior–caudal hyoid movements were precisely controlled. Following repositioning, the hyoid bone was fixed in place by the device.

### 2.3 Mandibular advancement

**FIGURE 2:**
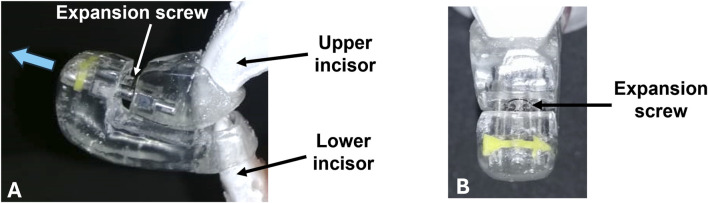
Mandibular advancement splint (MAS). **(A)** Side view of the MAS in which upper and lower incisors are inserted. The blue arrow indicates the direction of movement. The appliance is activated through an expansion screw that positions the lower incisor and mandible forward. **(B)** Frontal perspective of the appliance. The incorporated yellow arrow indicates the direction of screw activation.

A mandibular advancement splint (MAS) was custom made in-house based on plaster models of the rabbit’s maxilla (upper incisors) and mandible (lower incisors) (Figure 2). Alginate impression material was used to obtain a 3D impression of the models, which was then poured in white plaster. The MAS was constructed using cold-curing orthodontic acrylic resin (Vertex-Dental, AOPP2201000, shade 22, Netherlands) and incorporated an orthodontic expansion screw (Leone, A0890, Italy). The screw allowed for small gradual advancements of the mandible in the anterior direction.

The MAS was attached to the mandibular and maxillary incisors using glass ionomer luting cement (3M ESPE, self-curing, Germany). The MAS was fitted in such a way that the angle of mandibular advancement was 70° to the horizontal. By turning the screw in a clockwise direction, the mandible was displaced forward.

### 2.4 Pclose measurements

The collapsibility of the upper airway was quantified using Pclose, which represents the closing pressure of the upper airway. When the upper airway was open, the pressure detected at the level of the mask (Pm) was equivalent to the pressure at the level of the trachea (Pua). Subsequently, a negative pressure was applied to the upper airway using the syringe connected to the cranial trachea. Pua and Pm were carefully monitored until the point of deviation, indicating the closure of the upper airway. The minimal pressure value reached by Pm before diverging from Pua was defined as Pclose (Samaha et al., 2022). Relative to baseline, a more negative Pclose value indicates a less collapsible upper airway, while a more positive value indicates increased collapsibility.

### 2.5 Interventional protocol

The hyoid bone was re-positioned within the mid-sagittal plane in sequence along anterior, caudal, cranial, anterior-cranial (45°) (ant-cranial), and anterior-caudal (45°) (ant-caudal) directions by 0, 2 and 4 mm. At each hyoid displacement, the mandible was advanced by 0, 2 and 4 mm. Pclose was measured for each hyoid repositioning direction/increment and mandibular advancement level. Following each Pclose measurement, the system was re-opened to atmosphere (0 cmH_2_O) and then closed again, ready for the next measurement. The protocol was repeated three times.

### 2.6 Data and statistical analysis

All physiological signals were acquired using a Power Lab 16 channel acquisition system and recorded using Lab Chart 8 (ADInstruments Ltd., Colorado, United States). The primary outcome, ΔPclose, was averaged for each rabbit for all three runs. Group averaged data were represented as mean ± SD. For mandibular advancement alone, the average of all runs before applying hyoid repositioning in each direction was included in the analysis. In combined mandibular advancement and hyoid displacement analysis, the mandibular advancement values prior to the hyoid repositioning intervention in a particular direction were considered baseline for direct relevance.

A fixed effects linear model (IBM SPSS v24) was used to analyze the effect of the three independent variables (hyoid repositioning direction and increment and mandibular advancement increment) on the outcome variable ΔPclose (dependent variable). We used a fixed-effects linear model because the study involved repeated measures within the same animals, where each subject underwent all intervention conditions. This approach appropriately accounts for within-subject comparisons and isolates the effects of hyoid repositioning direction, hyoid increment, and mandibular advancement on Pclose. Subjects were included as a fixed effect to account for repeated measures. Interaction terms tested whether the effect of one factor depended on another. Pairwise comparisons with Bonferroni correction identified significant differences between the outcomes and their direction. Model assumptions (normality, homogeneity of variance) were checked and met. Statistical significance for all the analyses was inferred for p < 0.05.

## 3 Results

All 12 rabbits were included in the analysis. Each rabbit underwent three runs (i.e., three replicates per measurement), except for three animals in which only one run was performed per measurement due to physiological instability under anesthesia following the first run, which precluded further measurements. Accordingly, data from these single runs were used as the representative measurements for these animals.

**FIGURE 3:**
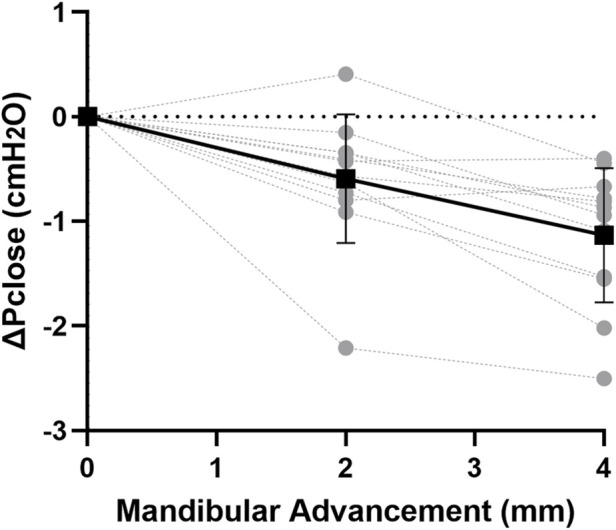
Change in closing pressure (ΔPclose) vs. mandibular advancement. Mandibular advancement alone caused a gradual decrease in ΔPclose with increasing increment. Individual rabbit (grey circles) and group mean values (black square) ± standard deviation (bars) are shown.

At baseline, Pclose was −4.2 ± 0.4 cmH_2_O.When mandibular advancement was applied alone, Pclose was significantly decreased at each increment (p < 0.001; Figure 3). On average, Pclose decreased by −0.6 and −1.1 cmH_2_O at mandibular advancement levels of 2 and 4 mm, respectively (Figure 3).

**FIGURE 4:**
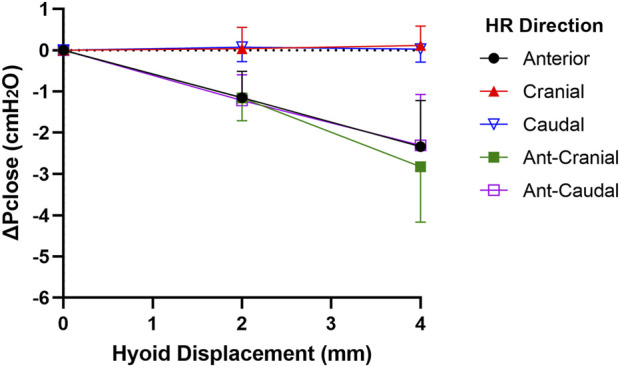
Change in closing pressure (ΔPclose) vs. hyoid repositioning (HR). Anterior (ant), ant-cranial and ant-caudal HR directions resulted in a gradual and similar decrease ΔPclose with each hyoid displacement. On the other hand, cranial and caudal HR directions had no significant effect on ΔPclose. Data are presented as mean group values (points) ± standard deviation (bars).

**FIGURE A1:**
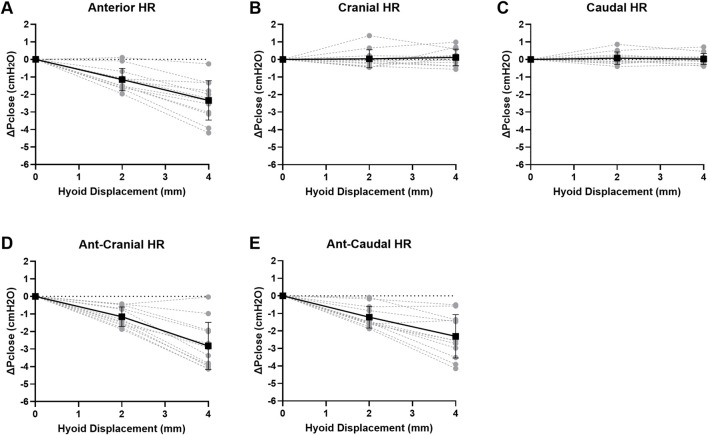
Change in closing pressure (ΔPclose) vs. hyoid repositioning (HR) in Individual Rabbits. Individual rabbit (grey circles) and mean ± SD rabbit data (black squares and bars) for **(A)** anterior (ant), **(B)** cranial, **(C)** caudal, **(D)** ant-cranial and **(E)** ant-caudal HR directions.

Pclose showed progressive decrease with each increment in hyoid displacement in the anterior, ant-caudal and ant-cranial directions, reaching on average −2.3 to −2.8 cmH_2_O at 4 mm (p < 0.001). Group data are shown in Figure 4, and individual rabbit data are presented in Figure A1. The decrease in Pclose was not statistically significant between all anterior-based directions at corresponding increments (p > 1.0). Pclose was not significantly altered when the hyoid was repositioned in cranial or caudal directions (p > 1.0; Figures 4, A1).

**FIGURE 5:**
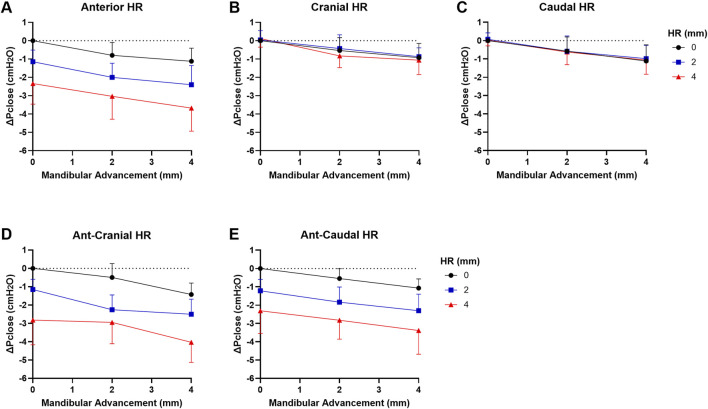
Change in closing pressure (ΔPclose) vs. mandibular advancement (MA) and hyoid repositioning (HR) combined. Increasing levels of MA at HR increments of 0, 2 and 4 mm are shown in **(A)** anterior, **(B)** cranial, **(C)** caudal, **(D)** ant-cranial, **(E)** ant-caudal directions. When MA is combined with HR, there was an additive effect on ΔPclose such that it decreased even further then when either intervention was applied alone for anterior, ant-cranial and ant-caudal directions **(A,D,E)**. However, ΔPclose did not significantly change with MA+HR for cranial and caudal directions compared to MA alone **(B,C)**. Data are presented as mean group values (points) ± standard deviation (bars).

The changes in ΔPclose when both mandibular advancement and hyoid repositioning were combined are shown in Figure 5. There was no significant interaction between mandibular advancement and hyoid repositioning on Pclose in any direction (p > 0.7). When mandibular advancement was combined with anterior, ant-cranial and ant-caudal hyoid repositioning directions, ΔPclose was more negative compared to when mandibular advancement was applied alone (p < 0.003; Figures 5A,D,E). For instance, a 4 mm mandibular advancement combined with ant-cranial hyoid displacement resulted in a mean ΔPclose of −4.0 cmH_2_O compared with −1.4 cmH_2_O at 4 mm mandibular advancement alone, and −2.8 cmH_2_O at 4 mm ant-cranial hyoid displacement alone (Figure 5). No significant differences were observed between anterior, ant-cranial and ant-caudal hyoid displacement effects on mandibular advancement induced ΔPclose outcomes (p > 1.0). Cranial and caudal hyoid displacement directions had no statistically significant effect on mandibular advancement (p > 1.0).

## 4 Discussion

This study has demonstrated that both graded mandibular advancement and hyoid repositioning in anterior-based directions, but not in cranial or caudal directions, independently decreased Pclose and hence reduced collapsibility of the upper airway. When mandibular advancement was combined with anterior-based hyoid repositioning, the effect was additive, resulting in further decrease in Pclose than when mandibular advancement was applied alone. These outcomes suggest that the effectiveness of mandibular advancement in treating OSA may be improved when combined with hyoid bone repositioning in anterior-based directions.

### 4.1 Mandibular advancement

Our findings are consistent with studies that showed reductions in upper airway collapsibility with mandibular advancement in humans (Bamagoos et al., 2019; Isono et al., 1995; Isono et al., 1997), which is likely to contribute to improved clinical outcomes in OSA, including reductions in apnea–hypopnea index (AHI), daytime sleepiness, and 24-h mean blood pressure (Farooq et al., 2025; Chen et al., 2025). Studies have also demonstrated that mandibular advancement enlarges the upper airway (Kuna et al., 2008; Sutherland et al., 2011; Amatoury et al., 2015; Degraeve et al., 2024) and can increase tissue stress/stiffness (Amatoury et al., 2016; Amatoury et al., 2015), factors that could mediate the observed reduced collapsibility. Computational fluid dynamics (CFD) modeling has also shown that mandibular advancement reduces intraluminal pressure and enhances flow along the length of the upper airway (Zhao et al., 2013). Furthermore, mandibular advancement can alter the tongue’s dilatory movements to potentially improve therapeutic response (Jugé et al., 2020). The effect of mandibular advancement on upper airway patency has also been partially related to the movement of the hyoid bone (Amatoury et al., 2016; Amatoury et al., 2015; Pae and Harper, 2021; Battagel et al., 1999).

When the mandible is advanced, the hyoid bone moves in an anterior/anterior-cranial direction (Amatoury et al., 2015; Battagel et al., 1999), which assists in redistributing the mandibular advancement load throughout the upper airway to enlarge and stiffen it (Amatoury et al., 2016; Amatoury et al., 2015). Indeed, a computational finite element model of the rabbit upper airway showed that mandibular advancement effects on upper airway soft tissue displacements were reduced when the hyoid was fixed compared to when it was free to move (Amatoury et al., 2016). Nonetheless, even with the hyoid fixed in the current study, relatively similar to surgical hyoid repositioning therapies for OSA, mandibular advancement can still reduce upper airway collapsibility. Mandibular advancement does not just impact the hyoid bone, but also alters the movement and stretch of the tongue (Jugé et al., 2020) and other muscles and connections to the upper airway, like the pterygomandibular raphe (Brown et al., 2020), so that increases in upper airway patency and stiffening of soft tissues can occur. However, if the hyoid remained mobile, it is possible that the effects of mandibular advancement would be greater (Amatoury et al., 2016).

### 4.2 Hyoid repositioning

The independent effects of hyoid displacement on upper airway collapsibility observed in this study are consistent with our previous experimental results in rabbits (Samaha et al., 2022). Anterior-based hyoid displacements progressively decreased upper airway collapsibility, while cranial and caudal hyoid displacements had no effect. Similar enhancements in upper airway patency through anterior and ant-caudal hyoid repositioning have also been observed in human and dog models (Van de Graaff et al., 1984; Rosenbluth et al., 2012; Yao et al., 2025).

We previously hypothesized that the apparent lack of impact of cranial or caudal hyoid repositioning on upper airway collapsibility may be associated with compression/stretching of tissues above (e.g., genioglossus, geniohyoid, hyoglossus, styloglossus, stylohyoid and palatoglossus muscles) and below (e.g., thyrohyoid membrane and ligament, and thyrohyoid, sternohyoid and omohyoid muscles) the hyoid bone (Samaha et al., 2022). It is likely that moving the hyoid cranially stretches upper airway tissues caudally, contributing to improved upper airway stability. However, the cranial hyoid movement compresses airway tissues cranially, leading to a more collapsible airway upstream. The opposite effect occurs with caudal repositioning. Consequently, the improvement in upper airway collapsibility in one segment is offset by a reduction in collapsibility in another, leading to an overall lack of change in collapsibility. In recent computational model simulations, we predicted that a decrease or lack of change in upper airway size with caudal/cranial hyoid repositioning likely contributes to the absence of change in Pclose observed with caudal/cranial hyoid repositioning experimentally (Salman and Amatoury, 2024).

### 4.3 Combined hyoid repositioning and mandibular advancement and clinical implications

To our knowledge, the combined effect of surgical hyoid repositioning and mandibular advancement has not been previously studied. The finding that combining hyoid repositioning in anterior-based directions with mandibular advancement leads to a more pronounced decrease in Pclose compared to either intervention alone is both novel and significant. For instance, the combination of mandibular advancement by 4 mm and hyoid ant-cranial repositioning by 4 mm yielded a 183% additional decrease in upper airway collapsibility compared to a 4 mm mandibular advancement alone. This combination is particularly impactful as upper airway tissues and dilator muscles are pulled in approximately the same direction by both interventions, which in turn likely enlarges the upper airway and stiffens the surrounding soft tissues. Thus, a treatment approach combining ant-cranial hyoid repositioning through hyomandibular advancement surgery, along with mandibular advancement, may improve airway patency in individuals who do not respond well to mandibular advancement alone. Additionally, combining surgical hyoid repositioning with mandibular advancement may reduce the excessive amount of mandible advancement required for effective OSA treatment, thereby potentially mitigating side effects such as temporomandibular or dental related pain, or dental/skeletal structural changes (Bartolucci et al., 2018; De Meyer et al., 2021). Further studies examining a combined hyoid repositioning and mandibular advancement therapy approach in humans are necessary to confirm these hypotheses.

### 4.4 Critique of methods

General limitations of the current study with hyoid repositioning alone and other interventions have been detailed previously (Samaha et al., 2022; Amatoury et al., 2014; Amatoury et al., 2015), and will be discussed briefly here.

#### 4.4.1 Rabbit model

A rabbit model was used for the current study, which we and others have repeatedly adopted in investigating upper airway physiology and mechanics with demonstrated similarity of rabbit upper airway outcomes to the human circumstance (Kirkness et al., 2003a; Amatoury et al., 2014; Brouillette and Thach, 1980; Amatoury et al., 2015; Amatoury et al., 2016; Kairaitis et al., 2006; Kairaitis et al., 2012; Olson et al., 1989; Kirkness et al., 2003b; Schiefer et al., 2020; Benderro et al., 2018; Serghani et al., 2024; Samaha et al., 2022). Although the rabbit’s craniofacial structure differs from that of humans and it possesses an overlapping soft palate and epiglottis, the general similarity of its upper airway structure makes it ideal for understanding concepts related to hyoid repositioning and mandibular advancement. Both interventions applied alone have shown comparable outcomes in the anesthetized rabbit to the sleeping or anesthetized human (Samaha et al., 2022; Amatoury et al., 2016; Amatoury et al., 2015; Salman and Amatoury, 2024), which provides us with confidence in the translatability of our combined intervention outcomes to the human. Furthermore, an advantage of the rabbit model is its mobile hyoid bone, which lacks fixed bony attachments like the human, a characteristic not found in other non-primates such as dogs, felines and rats (Amatoury et al., 2014; Amatoury et al., 2015). This allowed the hyoid to be readily repositioned in all directions and increments adopted in this study.

It is important to note that our rabbit model is not designed to replicate OSA but rather to simulate a healthy, well-controlled upper airway, which is one of the model’s major advantages to understanding fundamental mechanisms (see also below). OSA is a complex disorder influenced by multiple factors that require varied treatment approaches, including combination therapies (Carberry et al., 2018; Kairaitis et al., 2021). We propose that, in certain individuals with OSA, this combination of mandibular advancement and anterior-based hyoid repositioning could be key to successful treatment. However, future studies in humans are needed to identify which individuals would benefit most from this approach.

An isolated upper airway preparation was used in this study with rabbits deeply anaesthetized, as per previous preparations (Amatoury et al., 2014; Amatoury et al., 2015; Samaha et al., 2022). General anesthesia reduces upper airway muscle tone (Eastwood et al., 2002a; Hillman et al., 2010). This reduction is ideal for our study, as general anesthesia creates an upper airway model similar to sleep, particularly in terms of collapsibility (Eastwood et al., 2002b; Hillman et al., 2010). The ketamine and xylazine combination used in this study is extensively employed as an anesthetic agent in upper airway research (Amatoury et al., 2014; Amatoury et al., 2015; Rowley et al., 1996; Kairaitis et al., 2003; Kairaitis et al., 2006; Kirkness et al., 2003a). Xylazine induces central muscle relaxation and anesthesia (Green et al., 1981), while ketamine, in addition to providing sedation, counteracts any respiratory depression caused by xylazine, helping to maintain a relatively stable breathing pattern (Eikermann et al., 2012; Uzun et al., 2006). This combination is suitable for airway studies due to its ability to maintain near-passive airway characteristics, with upper airway muscle activity further reduced with the isolated upper airway in the current study, while preventing complete airway collapse (Mishima et al., 2020; Drummond, 1996). The initial step in understanding the passive response of the upper airway to combined hyoid bone repositioning and mandibular advancement is necessary and advantageous for using animal models. By removing factors associated with upper airway muscle activity and airflow, we can first understand how hyoid repositioning and mandibular advancement impact the upper airway alone, and then comprehend in the future how muscle activity and upper airway airflow may alter outcomes in an intact upper airway (Song and Pae, 2001; Kairaitis et al., 2012).

#### 4.4.2 Hyoid bone repositioning and mandibular displacements

The hyoid bone was fixed in the new position and unable to move with any additional load, including mandibular advancement. This set-up is likely relatively similar to hyoid repositioning surgeries, in which the hyoid bone is attached to the mandible or the thyroid cartilage. However, the degree of hyoid mobility following such surgeries remains unknown. During normal functioning, the hyoid moves in response to various active and passive loads. Indeed, the hyoid bone is displaced with mandibular advancement (Amatoury et al., 2015; Battagel et al., 1999). Experimentally, preserving hyoid mobility after displacement in all directions investigated in this study is challenging. However, additional research, such as with computational modeling, could explicitly explore how preserving hyoid mobility post-surgical repositioning may further enhance upper airway patency.

We did not investigate posterior hyoid repositioning, as this is not a viable surgical option and would likely worsen upper airway patency. However, examining the impact of a naturally more posterior hyoid position on mandibular advancement, as well as other anatomical variations, could be informative. Nevertheless, this is not feasible in animal or human models, since such repositioning would alter other properties (e.g., length/tension of tissues), and would require computational modeling (Salman and Amatoury, 2024), representing a potential avenue for future work.

A potentially useful metric would be to quantify hyoid position relative to the mandibular plane, as is commonly done in humans (Battagel et al., 1999; Costa e Sousa and dos Santos Gil, 2013), across different hyoid and mandibular displacement increments. The advantage of using a model based on healthy adult rabbits of the same species, gender, and similar age is that the hyoid and mandible are generally positioned similarly in each animal, including after hyoid repositioning. Nevertheless, we acknowledge that individual rabbits are not identical, and hyoid–mandibular plane distances could vary between animals. Measuring these distances using imaging could provide additional insight and represents a possible direction for future studies.

In the current study, we focused exclusively on forward mandibular displacements (i.e., mandibular advancements) combined with controlled hyoid repositioning. Conditions involving mandibular setback with hyoid repositioning in various directions were not examined, as these scenarios were beyond the scope of this work. Future studies could explore how different hyoid positions interact with mandibular setback to influence upper airway patency.

#### 4.4.3 Experimental control and validity

Hyoid repositioning magnitudes/directions (anterior, caudal, cranial, anterior–cranial, and then anterior–caudal displacements) and mandibular advancements were performed in a fixed order, rather than randomized. This may have introduced potential order effects (e.g., progressive muscle stretch or passive tension). However, we believe any such effects were minimal given the consistency of our findings, particularly the robust benefit of anterior-based directions in improving upper airway collapsibility with mandibular advancement. In addition, the full set of repositioning directions was completed before the next experimental run, which likely reduced potential carryover effects.

We did not conduct a power analysis to determine the sample size for this exploratory animal study prior to its implementation. Instead, we used sample sizes from similar previous rabbit model investigations, both our own and those conducted by others, as a guide (Amatoury et al., 2014; Amatoury et al., 2015; Samaha et al., 2022; Kairaitis et al., 2006; Kirkness et al., 2003a; Roberts et al., 1984). However, a *post hoc* power analysis revealed that for an alpha of 0.05, the power to detect differences in Pclose between increments of hyoid repositioning (for each direction) and mandibular advancement was at least 87%. The exception was the comparison between mandibular advancement of 2 mm and 4 mm, which had a power of 68%. Thus, the findings from this comparison should be interpreted with some caution.

Measurements were not blinded in the current study, which represent a potential limitation. Blinding was difficult to implement, as the hyoid repositioning and mandibular advancement interventions are inherently observable during data collection. However, all measurements were performed using objective instrumentation and standardized procedures, minimizing the potential for observer bias. Data analysis was also not blinded, which may have introduced additional bias. Nonetheless, each rabbit served as its own control, with all interventions applied sequentially and effects measured relative to baseline. This within-subject design reduces variability between animals and helps mitigate, but does not entirely eliminate, the potential for bias from unblinded analysis.

#### 4.4.4 Pclose quantification, translational relevance and complementary measures

Baseline Pclose in our rabbit model (i.e., when mandibular advancement and hyoid displacement = 0 mm) was −4.2 ± 0.4 cmH_2_O. Peak negative upper airway pressures during normal breathing in anesthetized rabbits average approximately −0.3 ± 0.05–0.6 cmH_2_O (Kairaitis et al., 2003), whereas in humans, breathing pressures during sleep range more widely, for instance, from approximately −1.3 to −7.3 cmH_2_O (Tong et al., 2019; Amatoury et al., 2018).

Collapsibility data are reported as changes in Pclose to minimize any potential inter-animal variability, as our primary focus is the relative change in Pclose with hyoid repositioning and/or mandibular advancement rather than absolute values. For context, in non-obese anesthetized (with muscle paralysis) human subjects without OSA, baseline Pclose was approximately −3.5 to −5 cmH_2_O, decreasing to around −12.5 to −15 cmH_2_O with manual mandibular advancement [lower jaw thrust, magnitude unknown; estimated from Figure 5 in Isono et al. (1997), corresponding to an ∼200–257% decrease (Isono et al., 1997)]. In sleeping participants with OSA, Pcrit (critical closing pressure) decreased from approximately 1.8 ± 3.9 to −4.0 ± 3.6 cmH_2_O with maximally comfortable mandibular advancement application [∼8 mm; calculated from Table 1 in Bamagoos et al. (2019)], a −5.8 cmH_2_O change (∼322% average decrease) (Bamagoos et al., 2019).

In the present study, mandibular advancement reduced from approximately −4.2 to −5.3 cmH_2_O at 4 mm mandibular advancement (∼26% reduction). To our knowledge no other studies apart from our previous work in a similar rabbit model (Samaha et al., 2022) have investigated upper airway collapsibility with hyoid repositioning. Although differences in experimental setup, protocol, species, and study populations (e.g., healthy vs. OSA), as well as measurement techniques, limit direct comparisons, the consistent direction and magnitude of change across studies demonstrate that mandibular advancement markedly reduces upper airway collapsibility, reinforcing the translational relevance of our findings.

While this study focused on upper airway collapsibility, as quantified by Pclose, additional outcomes, such as tissue stress and upper airway geometry, could provide complementary insights into airway mechanics. These measures were beyond the scope of the current study, but could be investigated in future work using computational modeling, imaging, or other approaches (Bekdache and Amatoury, 2024; Amatoury et al., 2015; Jugé et al., 2020). Incorporating such measures could help further elucidate how hyoid repositioning and mandibular advancement interact to influence upper airway patency.

### 4.5 Conclusion

This study has demonstrated that combining mandibular advancement with anterior-based hyoid bone repositioning leads to further reduction in upper airway collapsibility compared to either intevention applied alone. However, no significant effect was observed with cranial or caudal hyoid repositioning. These findings suggest that indivudals with OSA who do not respond adequately to mandibular advancement alone may benefit from a combined therapeutic approach involving both mandibular advancement and anterior-based surgical hyoid repositioning. Such a combined treatment strategy holds promise for enhancing the management of OSA in these individuals. Further research and clinical studies are necessary to validate these findings in humans and refine OSA treatment protocols for personalized patient care.

## Data Availability

The raw data supporting the conclusions of this article will be made available by the authors, without undue reservation.
